# Synergistic Antibacterial and Antibiofilm Activity of the MreB Inhibitor A22 Hydrochloride in Combination with Conventional Antibiotics against *Pseudomonas aeruginosa* and *Escherichia coli* Clinical Isolates

**DOI:** 10.1155/2021/3057754

**Published:** 2021-08-25

**Authors:** Anastasia Kotzialampou, Efthymia Protonotariou, Lemonia Skoura, Afroditi Sivropoulou

**Affiliations:** ^1^Department of Genetics, Development and Molecular Biology, School of Biology, Aristotle University of Thessaloniki, Thessaloniki 54124, Greece; ^2^Department of Microbiology, AHEPA University Hospital, S. Kiriakidi Str. 1, Thessaloniki 54636, Greece

## Abstract

In the era of antibiotic resistance, the bacterial cytoskeletal protein MreB is presented as a potential target for the development of novel antimicrobials. Combined treatments of clinical antibiotics with anti-MreB compounds may be promising candidates in combating the resistance crisis, but also in preserving the potency of many conventional drugs. This study aimed to evaluate the synergistic antibacterial and antibiofilm activities of the MreB inhibitor A22 hydrochloride in combination with various antibiotics. The minimum inhibitory concentration (MIC) values of the individual compounds were determined by the broth microdilution method against 66 clinical isolates of Gram-negative bacteria. Synergy was assessed by the checkerboard assay. The fractional inhibitory concentration index was calculated for each of the A22-antibiotic combination. Bactericidal activity of the combinations was evaluated by time-kill curve assays. The antibiofilm activity of the most synergistic combinations was determined by crystal violet stain, methyl thiazol tetrazolium assay, and confocal laser scanning microscopy analysis. The combined cytotoxic and hemolytic activity was also evaluated toward human cells. According to our results, *Pseudomonas aeruginosa* and *Escherichia coli* isolates were resistant to conventional antibiotics to varying degrees. A22 inhibited the bacterial growth in a dose-dependent manner with MIC values ranging between 2 and 64 *μ*g/mL. In combination studies, synergism occurred most frequently with A22-ceftazidime and A22-meropemen against *Pseudomonas aeruginosa* and A22-cefoxitin and A22-azithromycin against *Escherichia coli*. No antagonism was observed. In time-kill studies, synergism was observed with all expected combinations. Synergistic combinations even at the lowest tested concentrations were able to inhibit biofilm formation and eradicate mature biofilms in both strains. Cytotoxic and hemolytic effects of the same combinations toward human cells were not observed. The findings of the present study support previous research regarding the use of MreB as a novel antibiotic target. The obtained data expand the existing knowledge about the antimicrobial and antibiofilm activity of the A22 inhibitor, and they indicate that A22 can serve as a leading compound for studying potential synergism between MreB inhibitors and antibiotics in the future.

## 1. Introduction

The antibiotic resistance crisis has become one of the major threats of public health according to the World Health Organization (WHO) [1]. Globally, infections caused by multidrug resistant (MDR) Gram-negative bacteria are on the rise and responsible for high mortality and morbidity rates [2]. Among Gram-negative pathogens, carbapenem-resistant (CR) *Pseudomonas aeruginosa* and members of the *Enterobacteriaceae* family, especially extended-spectrum beta-lactamases (ESBLs)-producing and metallo-beta-lactamases (MBLs)-producing *Escherichia coli*, are associated with severe healthcare- and community-acquired infections, mainly in immunosuppressed and other vulnerable patients [3–6]. Today, a growing number of strains have been identified as resistant to essentially all commonly used antibiotics with a variety of resistance mechanisms including overexpression of efflux pumps, low membrane permeability, and target alterations [7]. Since several classes of conventional antibiotics cannot effectively treat infections caused by MDR *E. coli* and *P. aeruginosa*, the WHO has ranked the development of novel therapeutics against these pathogens as a critical priority [8].

Besides the emergence of multidrug resistance, the ability of those pathogens to colonize abiotic and biotic surfaces by forming biofilms provokes a serious concern regarding the chemotherapy of chronic nosocomial and medical device-associated infections [9]. Biofilm cells are reported to be 1000 times more resistant to antibiotics and the host's defense system than planktonic cells due to the protective polymeric matrix that restricts drug penetration and protects the pathogen from phagocytosis [10, 11]. It is reported that 65% of all microbial infections are associated with biofilm development [12].

The emergence of resistant pathogens and the associated loss of efficacy of many conventional drugs highlight the need for the development of novel antibiotics with alternative targets [13], as well as the development of new therapeutic strategies targeting biofilm formation and eradication [14]. Combinational antibiotic therapies have proven to be effective in the past, and nowadays they are the mainstay treatment for MDR bacterial infections. Consequently, it is an urgent need to further investigate synergistic interactions between novel antimicrobials and clinical antibiotics in combating the resistance crisis, but also preserving the efficacy of the antibiotics that are already in clinical use [15].

In recent years, components of the bacterial cytoskeleton are suggested as prospective drug targets [16, 17]. More specifically, the bacterial actin homolog MreB is reported as a potential antibiotic target [16–19] because it is conserved in most rod-shaped bacteria with a fundamental role in cell growth regulation, cell wall morphogenesis, cell division, cell polarity, and chromosome segregation [18, 20–24]. It has been reported that rod-shaped bacteria with several mutations in the MreB gene adopted a spherical phenotype followed by lysis, indicating the importance of MreB protein in the determination and maintenance of cell shape [17, 18]. Moreover, as an ATPase MreB plays a crucial role in the organization of multienzyme complexes essentials for murein synthesis [25] and regulates the localization of motility complexes in several bacterial strains [26]. Thus, the presence and functionality of MreB is essential for bacterial viability [18]. Besides the multifunctional character, another characteristic that makes cytoskeletal proteins promising antibiotic targets is the structural and functional differences with their eukaryotic analogs, which allows the development of specific bacterial inhibitors, reducing the possibility of toxic effects in mammalian cells [17]. Today, three components of the MreB structure have been recognized as possible antibiotic targets, including the nucleotide and A22 binding sites, which are essentials for the ATP-dependant polymerization [27, 28], and the interprotofilament interface of the protein, which is important for the double-filament formation [29]. Hence, compounds that could interfere with these components are promising antibiotics.

**Figure 1:**
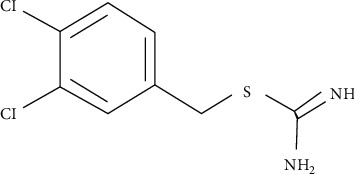
Chemical structure of A22 hydrochloride.

Natural and synthetic compounds targeting the prokaryotic cytoskeleton have long been introduced and used as research tools to study the multifunctional cytoskeletal proteins. Although many of those inhibitors have been reported as promising candidates for the development of novel antimicrobials, approved therapeutics are still not available [18]. Several studies have been done so far on the antibacterial activity of natural or chemical MreB inhibitors, including the most known and well-studied S-benzylisothiourea derivatives [16–18] and also bacterial toxins [29], indole-based compounds [30], and small protein regulators of the MreB assembly [18, 31]. S-(3,4-dichlorobenzyl)-isothiourea hydrochloride (A22 hydrochloride, A22) (Figure 1) is a reversible MreB inhibitor first described by Iwai et al. to induce spherical and anucleate cells in *E. coli* [32]. For many years, A22 has been the only known anti-MreB compound and primarily used in basic research to study the structure and function of MreB in several bacterial strains [17]. According to existing knowledge, A22 disrupts the rod shape of bacterial cells by impeding the ATP-dependent polymerization of MreB [27], but the precise mechanism of the drug has not been clearly understood. By targeting MreB [33], A22 impedes essential subcellular processes including cell wall synthesis, cell wall morphogenesis, cell division, and chromosome segregation [18, 20, 24]. Thenceforth, studies have shown the antibacterial activity of A22 against planktonic cells of several pathogens including *E. coli* and *P. aeruginosa* [34–37], as well as the inhibitory effect of A22 on *P. aeruginosa* biofilm formation [38], with minimal cytotoxic and genotoxic effects [37]. However, to our knowledge, no study has been reported about the activity of A22 on clinical *E. coli* biofilms and its efficacy to eradicate mature biofilms formed by Gram-negative clinical isolates. Moreover, the combined antibacterial, antibiofilm, cytotoxic, and hemolytic activity of A22 with conventional antimicrobials has not been examined so far.

The aim of this study was to investigate *in vitro* the antibacterial activity of A22 alone and in combination with antibiotics against a number of drug-sensitive as well as several MDR *P. aeruginosa* and *E. coli* clinical isolates. The combined effect of A22 on clinical bacterial biofilm formation and eradication was also examined. In addition, the cytotoxic and hemolytic profile of the combinations toward human cells was evaluated in order to examine the possibility of synergistic or reducing toxic effects.

## 2. Materials and Methods

### 2.1. Bacterial Strains, Culture Media, and Antimicrobial Agents

A total of 66 clinical isolates consisting of 30 *P. aeruginosa* and 36 *E. coli* recovered from patients hospitalized in AHEPA University Hospital were included in this study. Bacterial identification was performed with the VITEK^®^ 2 automated system (bioMérieux, Marcy I'Etoile, France). The standard strains of *P. aeruginosa* (NCIMB 12469) and *E. coli* (NCIMB 8879) were obtained from the National Collection of Industrial, Food, and Marine Bacteria (NCIMB, Scotland, United Kingdom) and were used as quality controls for the susceptibility tests. Bacteria were cultured on Mueller-Hinton agar II (MHA) and cation-adjusted Mueller-Hinton broth II (CAMHB) (Sigma-Aldrich, St. Louis, MO, USA) and were maintained at −80°C in 50% glycerol. Fresh subcultures from glycerol stocks were prepared before each experiment. A22 was obtained from Sigma-Aldrich (Product Number SML0471-10 MG, St. Louis, MO, USA), diluted in absolute ethanol, and stored at −20°C. Amikacin (AMK) (Vocate S.A.), ampicillin-sulbactam (A/S) (Pfizer Hellas S.A.), azithromycin (AZM) (Vocate S.A.), cefoxitin (CFX) (Vianex S.A.), ceftazidime (CAZ) (Vocate S.A.), ciprofloxacin (CIP) (Cooper S.A.), colistin (CL) (Norma Hellas S.A.), gentamicin (GEN) (Sopharma AD), and meropenem (MERO) (Vianex S.A.) were used in this study as conventional antibiotics. Stock solutions were prepared according to the Clinical and Laboratory Standards Institute^®^ (CLSI) recommendations [39] and stored at the optimum temperature for each drug.

### 2.2. Determination of Minimum Inhibitory Concentration (MIC)

Minimum inhibitory concentrations (MICs) of A22 and antibiotics were determined by the broth microdilution method according to CLSI guidelines [39]. The appropriate antibiotics were selected for testing on each bacterial strain according to CLSI criteria. In brief, serial twofold dilutions (256–0.007 *μ*g/mL) of the tested compounds were prepared on CAMHB. Fifty microliters of each dilution was transferred in the appropriate well of a 96-well microplate (SPL Life Sciences Co., Ltd., Korea). Each well was inoculated with 50 *μ*L of cells derived from a logarithmic phase broth culture in order to achieve a final concentration of 5 × 10^5^ colony-forming units (cfu)/mL. The plates were incubated at 37°C for 16–20 h. The MIC was defined as the lowest concentration of A22 or antibiotic resulting in no visible bacterial growth. The susceptibility breakpoints for all conventional drugs were interpreted according to CLSI breakpoint recommendations [39]. The MIC values were obtained from three independent experiments.

### 2.3. Checkerboard Assay

The combined antimicrobial activity of A22 with conventional antibiotics was determined by the broth microdilution checkerboard technique as previously described with minor modifications [40]. Briefly, A22 was twofold diluted in CAMHB (1/32–2 × MIC) down the columns of a 96-well microplate, while the antibiotic of the combination was diluted along the rows of the plate (1/128–2 × MIC). Each well was inoculated with bacterial suspension to achieve a final concentration of 5 × 10^5^ cfu/mL. After incubation at 37°C for 16–20 h, the MIC of each compound in the combination was identified as the lowest concentration completely inhibiting the visible bacterial growth. Based on the obtained data, the fractional inhibitory concentration index (FICI) was calculated for each combination according to the follow formula: FICI = MIC of A22 in combination/MIC of A22 alone + MIC of antibiotic in combination/MIC of antibiotic alone. FICI results were interpreted as synergistic (FICI ≤ 0.5), additive/indifferent (FICI = 0.5–4), and antagonistic (FICI°>°4) [41]. FICI values were assessed in 15 *E. coli* and 15 *P. aeruginosa* clinical isolates. Most of the isolates were resistant or intermediate resistant to the antibiotic of the combination (Tables S1 and S2). The presented data are the results obtained from three independent experiments.

### 2.4. Time-Kill Curve Analyses

In order to evaluate the killing kinetics of A22 and to investigate if synergy exerted lethal activity, the time-kill curve method was performed according to the National Committee for Clinical Laboratory Standards (NCCLS) guidelines [42]. Time-kill analyses were performed only on the combinations found to be synergistic in a percentage higher than 50% based on the checkerboard results (A22 and CAZ, MERO, GEN, CIP for *P. aeruginosa*; A22 and CFX, AZM for *E. coli*) against six randomly selected clinical isolates, comprising three strains of *P. aeruginosa* and *E. coli* each. Bacteria were subcultured in CAMHB until the logarithmic phase and diluted in 5 mL CAMHB to achieve a concentration of 5 × 10^5^ cfu/mL. A22 and antibiotics were added as single agents or in combination at concentrations to allow for assessment of synergy (based on the MIC for each strain). A drug-free growth control was included in all tests. All samples were incubated at 37°C in a shaker incubator (150 r.p.m.). At indicated time points, 100 *μ*L of each sample was serially diluted in phosphate-buffered saline (PBS) and plated on MHA plates for colony counting (cfu/mL). Time-kill curves were analyzed by plotting log_10_(cfu/mL) versus time. The detection limit was 1-log_10_(cfu/mL). Bacteriostatic and bactericidal activity were defined as <3-log_10_ and ≥3-log_10_ reduction in cfu/mL, respectively, relative to the initial inoculums. Synergy was defined at 24 h as a ≥2-log_10_ decrease in cfu/mL between the combination and the most effective single compound [42]. A combination was considered as synergistic bactericidal when it obtained a reduction of at least 2-log_10_(cfu/mL) in relation to the most active agent and a reduction of at least 3-log_10_(cfu/mL) in relation to the initial inoculum both at 24 h. Each experiment was repeated three times, and each assay was performed in duplicate. The results of a representative experiment with duplicate colony-forming determinations are presented.

### 2.5. Inhibition of Biofilm Formation

The combinations that showed high synergistic effects (>80%) against planktonic cells were chosen to be examined for their antibiofilm efficacy against three strong biofilm-forming clinical isolates of *P. aeruginosa* and *E. coli* (A22 and CAZ, MERO for *P. aeruginosa*; A22 and AZM, CFX for *E. coli*). The biofilm formation was determined by quantifying the biofilm biomass (crystal violet assay, CV) and the viable cells within the matrix [MTT assay, 3-(4,5-dimethylthiazol-2-yl)-2,5-diphenyltetrazolium bromide]. Briefly, overnight grown cultures were diluted in CAMHB supplemented with 2% glucose to achieve a concentration of 10^7^ cfu/mL and incubated statically in 96-well flat-bottom microplates with or without the addition of sub-MIC to MIC A22 or/and selected antibiotics (1/16–1 × MIC) at 37°C for 24 h. To quantify biofilm biomass, planktonic bacteria were discarded and the wells were rinsed twice with PBS, air-dried for 30 min, and stained with 0.1% (w/v) crystal violet solution for 10 min at room temperature. Excess stain was discarded, the wells were washed twice with water and air-dried for 1 h. Crystal violet bound to the biofilm matrix was extracted with 95% (v/v) ethanol, and the absorbance was measured at 570 nm using a microplate autoreader (BIO-TEK instruments, USA).

The metabolic activities of viable biofilm cells were assessed by the MTT assay. After 24 h of incubation, nonadherent bacteria were rinsed twice with PBS, and 0.5 mg/mL MTT reagent (Sigma-Aldrich, St. Louis, MO, USA) was added to the wells following 3 h incubation at 37°C in the dark to allow the formation of formazan crystals. After incubation, the supernatant of the wells was discarded, crystals were solubilized by the addition of dimethyl sulfoxide (DMSO) (Sigma-Aldrich, St. Louis, MO, USA), and the absorbance was determined at 570 nm. The percentage of biofilm inhibition and cell viability was expressed compared with the untreated controls (100% biofilm formation and 100% cell viability). Each assay was performed in triplicate.

### 2.6. Biofilm Disruption Assay-Confocal Laser Scanning Microscopy (CLSM)

To determine the effect of A22 and antibiotics on mature biofilms, MTT and CLSM microscopy studies were conducted. Bacteria cells derived from a mid-log phase culture were inoculated in CAMHB supplemented with 2% glucose to achieve a concentration of 10^8^ cfu/mL. One hundred microliters of the cells was transferred to the wells of a 96-microplate and incubated statically at 37°C for 24 h to allow biofilm formation. After incubation, the supernatant was discarded, wells were rinsed with PBS, and fresh medium containing sub-MICs to upper-MICs of A22 and/or selected antibiotics was poured gently without disturbing the adhered cells and incubated for another 24 h. The metabolic activities of viable biofilm cells were determined by the MTT assay as described in the biofilm inhibition assay.

For the dispersion assay of the preformed biofilms after exposure to A22 and antibiotics, CLSM imaging was performed. Briefly, sterilized cover slips were immersed in CAMHB+2% glucose, inoculated with 10^8^ cfu/mL bacterial cells in a 35 mm Petri plate, and incubated statically at 37°C for 24 h. After incubation, the cover slips were washed with PBS and immersed in fresh medium containing A22 and selected antibiotics at concentrations based on the MIC and incubated for another 24 h. Biofilms on the cover slips were rinsed twice with PBS and stained with 0.1% acridine orange solution for 1 min in the dark. Excess dye was removed with PBS, and the slips were air-dried, mounted with antifade solution, and visualized under CLSM (Model LSM780; Carl Zeiss AG, Munich, Germany) equipped with an excitation filter range of 515–560 and magnification at 10×. The CLSM images were analyzed using ZEN2011 software.

### 2.7. Evaluation of Combined Cytotoxic and Hemolytic Activity In Vitro

Human peripheral blood mononuclear cells (PBMCs), polymorphonuclear neutrophils (PMNs), and red blood cells (RBCs) were isolated from the peripheral blood of healthy donors (*n* *=* 3). Experiments were performed in accordance with the Declaration of Helsinki (7^th^ revision, 2013). All donors gave their written consent.

#### 2.7.1. PBMCs and PMNs Isolation

PBMC and PMN cells were isolated from whole heparinized blood by Lymphosep (Lymphocyte Seperation Media^CE^; Biowest, France) density gradient centrifugation at 400 × *g* at room temperature for 30 min according to the manufacturers' protocol. After centrifugation, the mononuclear band located between Lymphosep and blood plasma was transferred carefully to another centrifuge tube and washed three times with Dulbecco's phosphate-buffered saline (DPBS; Biowest, France) by centrifugation for 10 min at 300 × *g* at room temperature. The PMN and erythrocyte pellet formed under the Lymphosep layer were also transferred to a separate tube where the RBCs were lysed twice with ACK lysis buffer (150 mM NH_4_Cl, 10 mM KHCO_3_, 0.1 mM Na_2_EDTA, pH 7.2–7.4). The remaining PMNs were washed three times with DPBS by centrifugation for 10 min at 300 × *g* at room temperature. After the third wash, both PBMCs and PMNs were resuspended in 2-3 mL of RPMI-1640 media (Biowest, France) supplemented with 10% fetal bovine serum (FBS; Biowest, France). Cells were counted with 0.4% (w/v) trypan blue solution (Sigma-Aldrich, St. Luis, MO, USA) in a hemocytometer.

#### 2.7.2. Evaluation of Cytotoxicity

The cytotoxic activity of A22, antibiotics, and their combinations toward PBMC and PMN cells was determined by the standard MTT proliferation assay with minor modifications [43]. Briefly, cells were seeded in 96-well plates (∼1 × 10^5^ cells/well in 100 *μ*L final volume) and exposed to various concentrations of A22, antimicrobial compounds, and their mixtures for 24 h at 37°C and 5% CO_2._ Negative (0% cell viability) and positive (100% cell viability) controls were prepared with 100 *μ*L of complete RPMI-1640 medium and 100 *μ*L of cell suspension without the addition of drugs, respectively. After the treatment, 10 *μ*L of MTT solution (5 mg/mL diluted in DPBS) was added to each well and incubated in the dark for 4 h at 37°C and 5% CO_2_. After incubation, the plates were centrifuged at 1000 × *g* for 10 min, the supernatants were discarded, and 100 *μ*L of DMSO was added to each well to solubilize formazan crystals. Finally, the optical density (OD) was measured at 570 nm. The percentage of cell viability was calculated by the following formula:

(1) $$\begin{array}{c} \text{Viability}\left(\%\right)=\frac{\left({\text{OD}}_{\text{sample}}-{\text{OD}}_{0\%\text{ cell viability}}\right)}{\left({\text{OD}}_{100\%\text{ cell viability}}-{\text{OD}}_{0\%\text{ cell viability}}\right)}\times 100\%. \end{array}$$

The experiments were repeated at least three times, and each experiment was performed in triplicate.

#### 2.7.3. Hemolytic Assay

The hemolytic activity of A22, antibiotics, and their combinations was determined as previously described with some modifications [44]. Briefly, whole blood collected in K2EDTA BD Vacutainer^®^ tubes (BD, USA) was diluted with cooled DPBS in a ratio of 1 : 3 and centrifuged for 8 min at 700 × *g* and 4°C. RBCs were washed three times with DPBS by centrifugation under the same conditions. The sedimented RBCs were diluted in DPBS to obtain a 0.5% (v/v) RBC suspension, and an aliquot of 100 *μ*L of this solution was added to each well of a 96-well microplate already containing 100 *μ*L of twofold serially diluted A22 or/and antibiotics to reach a final volume in each well of 200 *μ*L and incubated for 1 h at 37°C and 5% CO_2_. Negative (0% hemolysis) and positive (100% hemolysis) controls were prepared by mixing 100 *μ*L RBC suspension with 100 *μ*L DPBS or 100 *μ*L Triton X-100 10% (v/v), respectively. After incubation, the plates were centrifuged at 1000 × *g* for 10 min, 100 *μ*L aliquots of the supernatants were transferred to clear microplates, and the OD was measured at 405 nm. The percentage of hemolysis was calculated by the following equation:

(2) $$\begin{array}{c} \text{Hemolysis}\left(\%\right)=\frac{\left({\text{OD}}_{\text{sample}}-{\text{OD}}_{\text{negative control}}\right)}{\left({\text{OD}}_{\text{positive control}}-{\text{OD}}_{\text{negative control}}\right)}\times 100\%. \end{array}$$

The experiments were repeated three times, and each assay was performed in triplicate.

### 2.8. Statistical Analysis

All assays were performed at least three times, and the results were expressed as means ± standard deviation (SD). To determine statistically significant differences, an analysis of variance (ANOVA) was performed followed by Tukey's multiple comparisons tests using the software package GraphPad Prism 8.0.1 (GraphPad, Inc., USA). The *p* value < 0.05 was considered statistically significant.

## 3. Results

### 3.1. Antimicrobial Susceptibility Test

Antimicrobial resistance patterns of the studied isolates and MIC ranges of the tested compounds are shown in Table 1. According to the obtained results, *P. aeruginosa* and *E. coli* isolates were resistant to selected antibiotics to varying degrees. The highest percentages of resistance were observed to meropenem and to ampicillin-sulbactam in *P. aeruginosa* and *E. coli* isolates, respectively. According to the MIC values of the conventional antibiotics, the most active agents were colistin for *P. aeruginosa* and meropenem for *E. coli*. A22 presented almost identical inhibitory activity against *P. aeruginosa* and *E. coli* strains, with MIC values ranging between 2–64 and 4–64 *μ*g/mL, respectively. In both strains, MIC of A22 was independent of the isolates' drug resistance profile (Tables S1 and S2). The MIC values of the antibiotics against the standard strains were within the CLSI-approved range.

### 3.2. Checkerboard Assay

The results of the checkerboard assays and the calculated FICIs of the combinations against the clinical isolates of *P. aeruginosa* (*n* *=* 15) and *E. coli* (*n* *=* 15) are summarized in Table 2. In both strains, A22 and antibiotics combinations showed varying degree of synergy. With an FICI value of ≤0.5 as borderline, synergistic effects were mostly seen with A22 + CAZ, A22 + MERO, A22 + GEN, and A22 + CIP combinations against *P. aeruginosa* (87, 80, 73, and 53%, respectively). Interestingly, for *E. coli* isolates, the combination of A22 with CFX and AZM exerted strong synergistic effects in almost all 15 tested isolates (100 and 93% synergy, respectively). An additive interaction was observed most frequently when A22 was combined with AMK and CL against *P. aeruginosa* and with MERO, AMK, CIP, and A/S against *E. coli*. No antagonism was observed in any of the tested combinations.

### 3.3. Time-Kill Kinetics

**Figure 2:**
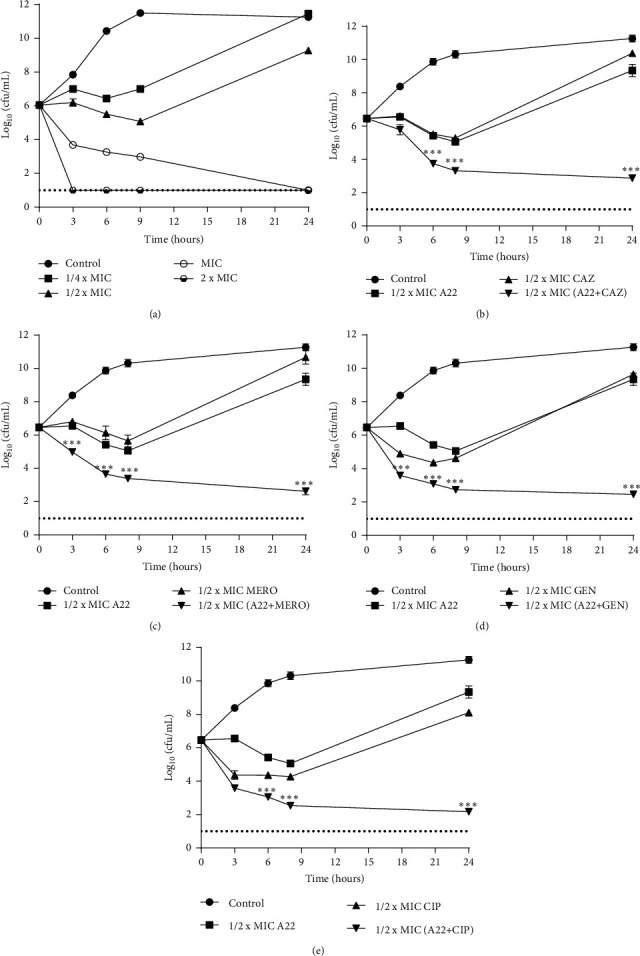
Time-kill assays for three clinical isolates of *P. aeruginosa* after treatment with (a) A22 alone at 1/4–2 × MIC and (b) A22 and ceftazidime (CAZ), (c) A22 and meropenem (MERO), (d) A22 and gentamicin (GEN), and (e) A22 and ciprofloxacin (CIP) at 1/2 × MIC. The results of a representative experiment with duplicate colony-forming determinations are presented. The error bars indicate the standard deviations between isolates. The limit of detection (1-log_10_(cfu/mL)) is indicated by the dash lines. ^∗∗∗^ = *p* < 0.001: significances between the combinations and the most active agent monotherapy. Two-way ANOVA with post hoc Tukey's comparisons.

**Figure 3:**
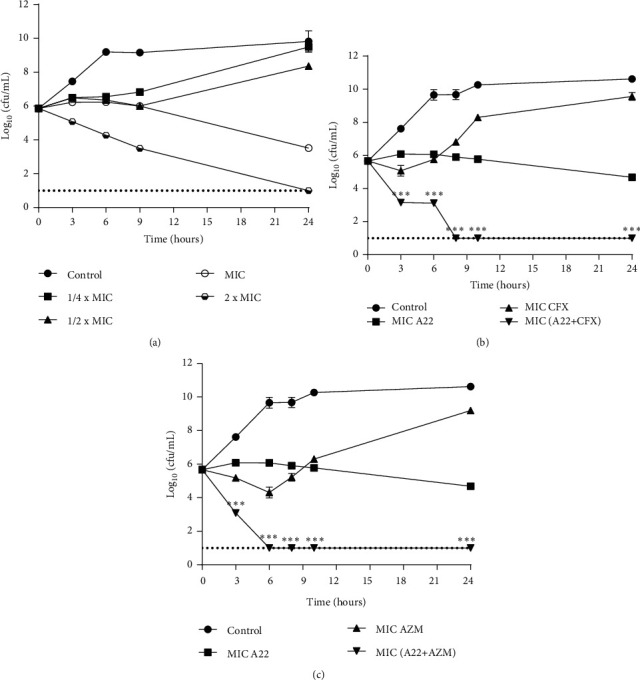
Time-kill assays for three clinical isolates of *E. coli* after treatment with (a) A22 alone at 1/4–2 × MIC and (b) A22 and cefoxitin (CFX) and (c) A22 and azithromycin (AZM) at 1 × MIC. The results of a representative experiment with duplicate colony-forming determinations are presented. The error bars indicate the standard deviations between isolates. The limit of detection (1-log_10_(cfu/mL)) is indicated by the dash lines. ^∗∗∗^ = *p* < 0.001: significances between the combinations and the most active agent monotherapy. Two-way ANOVA with post hoc Tukey's comparisons.

Based on the time-kill curve results, A22 monotherapy showed a dose-dependent killing effect against *P. aeruginosa* and *E. coli* clinical isolates. In both bacteria, the sub-MICs of A22 (1/4–1/2 × MIC) caused either an initial lag in growth or reduction in viable cells within 9 h and regrowth occurred to various levels by 24 h (Figures 2(a) and 3(a), Table 3). A22 exerted bactericidal activity against *P. aeruginosa* within 6 h and 3 h at 1 × MIC and 2 × MIC, respectively (Figure 2(a), Table 3). For *E. coli* isolates, A22 showed bacteriostatic activity at 1 × MIC, and a ≥3-log_10_ killing was determined in higher concentrations (2 × MIC) within 24 h (Figure 3(a), Table 3).

In combination time-kill studies, as shown in Figures 2(b)–2(e) and 3(b) and 3(c), synergism was observed with all expected combinations against studied clinical isolates of *P. aeruginosa* and *E. coli*. Significant synergistic interactions of A22 with ceftazidime, meropenem, gentamicin, and ciprofloxacin combinations were documented on *P. aeruginosa* isolates. Combinations of A22 with CAZ, MERO, GEN, and CIP at 1/2 × MIC revealed a >6-log_10_ decrease of colony counts after 24 h compared with the most active single agent (*p* < 0.001) (Figures 2(b)–2(e), Table 4). Particularly, the above combinations reduced the viable cell number of *P. aeruginosa* approximately 4-log_10_ after 24 h compared with the initial inoculums, suggesting a bactericidal synergistic effect (Figures 2(b)–2(e), Table 4). Similarly, synergism was observed in *E. coli* when A22 was combined with cefoxitin and azithromycin at 1 × MIC (Figures 3(b) and 3(c)). As presented in Table 4, the above synergistic combinations exerted lethal activity by a >4-log_10_ reduction of viable cells in relation to initial inoculum at 24 h.

### 3.4. Inhibition of Biofilm Formation

**Figure 4:**
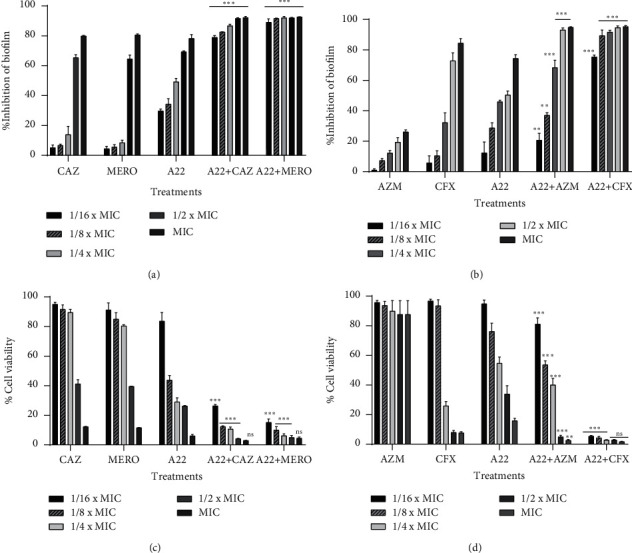
Effect of A22 in combination with antibiotics on *P. aeruginosa* and *E. coli* biofilm formation and cell viability. Inhibition of biomass formation quantified by the CV assay at 24-h treatment with sub-MICs to MICs of (a) A22 and ceftazidime (CAZ), meropenem (MERO) against 3 strong biofilm-producing *P. aeruginosa* clinical isolates and (b) A22 and azithromycin (AZM), cefoxitin (CFX) against 3 strong biofilm-producing *E. coli* clinical isolates. The metabolically active biofilm-producing cells were measured by the MTT assay on (c) *P. aeruginosa* and (d) *E. coli* biofilms. The percentage of biofilm inhibition and cell viability was expressed compared with the untreated controls (100% biofilm formation and 100% cell viability, respectively). Each assay was performed in triplicate. The error bars indicate the standard deviations. ns = nonsignificant, ^∗∗^ = *p* < 0.01, ^∗∗∗^ = *p* < 0.001: significances between the combinations and the most active agent monotherapy.

The inhibitory effects of A22 and antibiotics on biofilm formation are shown in Figure 4. A22 inhibited the biofilm formation of each three strong biofilm-forming clinical isolates of *P. aeruginosa* and *E. coli* at 24 hours in a concentration-dependent manner. 1/4 × MIC A22 resulted approximately in 50% inhibition of biomass formation in both strains. All of the studied antibiotics showed a poor antibiofilm activity and only at higher concentrations were able to inhibit the biofilm biomass formation (Figures 4(a) and 4(b)). As it was expected, in *P. aeruginosa* isolates, when A22 was used along with CAZ and MERO at sub-MIC combinations, biofilm biomass was reduced by 80% even at the lowest concentration of the combination (1/16 × MIC), showing a significantly enhanced antibiofilm activity (*p* < 0.001) (Figure 4(a)). Similarly, on *E. coli* isolates, a significant reduction of biofilm biomass was observed in the combinations of A22 with AZM and CFX compared with the most active agent (*p* < 0.001) (Figure 4(b)). MTT assays confirmed the crystal violet results (Figures 4(c) and 4(d)). The metabolically active biofilm-producing cells were measured to be 30% at 1/2 × MIC A22 in both strains. The combinations of A22 with MERO, CAZ and CFX, AZM against *P. aeruginosa* and *E. coli*, respectively, showed a statistically significant reduction in viable cells compared with the most active compound of the combinations (*p* < 0.001).

### 3.5. Biofilm Eradication-CLSM Analysis

**Figure 5:**
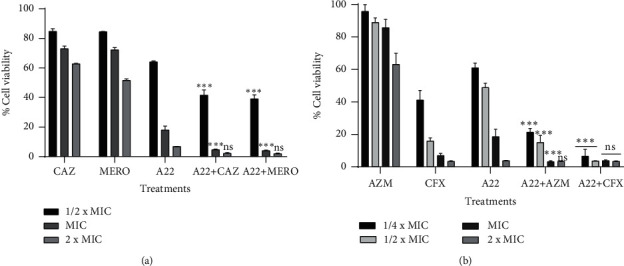
Effect of A22 in combination with antibiotics on *P. aeruginosa* and *E. coli* clinical preformed biofilms. Mature biofilms of (a) *P. aeruginosa* and (b) *E. coli* were treated for 24 h with sub-MICs to upper-MICs of A22, ceftazidime (CAZ), meropenem (MERO), and A22, azithromycin (AZM), cefoxitin (CFX), respectively. The percentage of cell viability was determined by the MTT assay and expressed compared with the untreated controls (100% cell viability). Each assay was performed in triplicate. The error bars indicate the standard deviations. ns = nonsignificant, ^∗∗∗^ = *p* < 0.001: significances between the combinations and the most active agent monotherapy.

**Figure 6:**
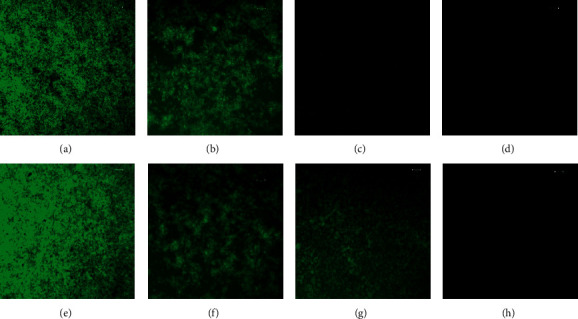
Representative CLSM microscopy images after acridine orange staining, showing the eradiation effects of A22 combined with antibiotics on *P. aeruginosa* and *E. coli* mature (preformed) biofilms: (a and e) untreated *P. aeruginosa* and *E. coli* controls, respectively; (b–d) *P. aeruginosa*-treated biofilms with MIC A22 alone or in combination with CAZ and MERO, respectively; and (f–h) *E. coli*-treated biofilms with 1/4 × MIC A22 alone or in combination with AZM and CFX, respectively. Scale bars: 50 *μ*m.

The eradiation efficacy of A22 and antibiotics on preformed biofilms was determined by MTT assay and through CLSM microscopy (Figures 5 and 6). As shown in Figure 5, A22 at MIC resulted in approximately 80% reduction of viable cells after 24 h of exposure in both *P. aeruginosa* and *E. coli* mature biofilms. The conventional antibiotics except cefoxitin, even at the highest tested concentration (2 × MIC), exhibited poor bactericidal activity, resulting in approximately 50% reduction of viable cells. However, synergistic combinations in all cases and tested concentrations were reported to be more active (Figures 5(a) and 5(b)). In addition, combinations were found to be very effective in disrupting the biomass of preformed biofilms. The CLSM images indicated major reduction of biofilm biomass and a great disruption in the mature biofilm architecture (Figure 6).

### 3.6. Combined Cytotoxic Activity

**Figure 7:**
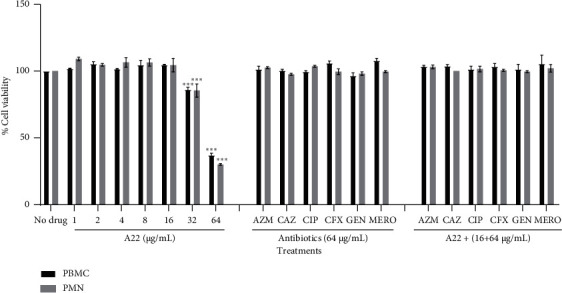
Effect of A22, monotherapy and in combination with antibiotics, on viability of human PBMCs and PMNs. The percentage of cell viability was assessed by the MTT assay and expressed compared with the untreated control (no drug, 100% viability). Data represent the means ± SD of three experiments. Each assay was performed in triplicate. ^∗∗∗^ = *p* < 0.001: significance was compared with the untreated control.

To determine the cytotoxicity of synergistic combinations, the MTT test was performed on human PBMC and PMN cells. As it was expected, antibiotics were nontoxic toward both PBMC and PMN cells at concentrations up to 64 *μ*g/mL. A22 caused a statistically significant decrease in cell viability (*p* < 0.001) only at the higher tested concentrations (32–64 *μ*g/mL) (Figure 7). A22 was combined with selected antibiotics in certain concentrations, which have been identified as antimicrobial and antibiofilm synergistic in the present study. No enhancement of the cytotoxic effect was observed in any of the combinations (Figure 7).

### 3.7. Combined Hemolytic Activity

The effect of A22 alone and in combination with antibiotics on human RBCs was studied. As presented in Table 5, the ability of A22 to induce hemolysis was either nonexistent or insignificant (<1.5%) even at the maximum tested concentration (256 *μ*g/mL). Similarly, the tested antibiotics were nonhemolytic at concentrations up to 256 *μ*g/mL. No case of enhancement of the hemolytic activity regarding combination treatments was detected.

## 4. Discussion

In recent years, the bacterial cytoskeletal protein MreB has emerged as a potential drug target in the battle of fighting antibiotic resistance and development of novel antimicrobial drugs. Among MreB inhibitors, A22 has been reported as a lead compound with potent antibacterial and antibiofilm activity [18], but its combined activity with other antimicrobials has not been characterized. Based on the promising background and the desirable properties of this drug, our study was dedicated to examining potential synergism between A22 and clinical used antibiotics against both planktonic and biofilm forms of MDR Gram-negative bacteria, considering their hemolytic and cytotoxic activity.

The antibacterial effect of A22 was determined against 66 clinical isolates of Gram-negative bacteria. A22 presented almost identical MIC range against *P. aeruginosa* and *E. coli* clinical isolates, and the obtained results are comparable with those of previous studies in that A22 could successfully inhibit the growth of several pathogens including *P. aeruginosa* and *E. coli* strains [32, 34–37, 45–47]. We found that 77.4% and 67.5% of the *P. aeruginosa* and *E. coli* isolates, respectively, obtained MIC values ≤ 16 *μ*g/mL (Tables S1 and S2). These findings corroborate previous reports in which A22 presented higher MIC values against *E. coli* isolates in relation to other bacterial strains [37, 48].

In both *P. aeruginosa* and *E. coli* strains, the MIC of A22 was independent of the isolates' resistance profile to conventional antibiotics. *P. aeruginosa* showed a high percentage of resistance to carbapenems (81%), cephems (42%), aminoglycosides (48%), and fluoroquinolones (65%). Similarly, most of the *E. coli* isolates exhibited high rates of resistance to beta-lactam antimicrobials (95%), including cephems, macrolides (89%), and fluoroquinolones (79%), while remaining variably susceptible to meropenem (35%) and amikacin (35%). As in our study, several reports demonstrated that CR *P. aeruginosa* is isolated more commonly from patients than CR *E. coli* [49, 50]. According to the CLSI breakpoints, the most active agent was colistin and meropenem for *P. aeruginosa* and *E. coli* isolates, respectively. Many reports suggest that colistin remains the most active and salvage agent in the treatment of severe infections caused by CR *P. aeruginosa* [4]. However, its safety is limited by high rates of nephrotoxicity [51]. Interestingly, many *E. coli* and *P. aeruginosa* isolates with low resistance profile to conventional antibiotics had equal or higher MIC values of A22 compared with drug-resistant ones. Further experiments involving more bacterial strains are clearly required to better investigate and conclude about any correlation between the MIC of A22 and the antibiotic resistance pattern of clinical isolates.

Combination antibiotic therapy represents an attractive therapeutic approach for the treatment of MDR-caused infections [52]. Combination therapy usually requires lower doses of the individual compounds, leading to synergistic effects with low risk of resistance development. A number of reports have demonstrated the effectiveness of antibiotic combinations against MDR Gram-negative bacteria [53]. Since no reports have been documented on the interaction of A22 with conventional drugs against Gram-negative clinical isolates, we studied several combinations against planktonic cells by the widely used checkerboard method. Interestingly, we found that A22 exhibited great synergistic growth-inhibitory effects with various antibiotics and no antagonism was observed with any of the combinations. Our results are in accordance with those of a previous study in which the synergistic effects of an A22-related benzylisothiourea derivative named “C2” with various antibiotics against *P. aeruginosa* planktonic cells have been observed. The authors concluded that C2 enhanced the antimicrobial activity of conventional antibiotics, but the FICI values of the combinations had not been evaluated [35].

In our study, the synergistic effects were strain-dependent, mostly seen with A22-ceftazidime and A22-meropemen against *P. aeruginosa* and A22-cefoxitin and A22-azithromycin against *E. coli,* even though most of the isolates were resistant to the antibiotic of the combination. Several combinational *in vitro* studies designed by the checkerboard method have demonstrated synergistic interactions against MDR pathogens even when the bacteria are resistant to the individual antimicrobials [54]. The precise mechanism by which A22 interacts with MreB is not well characterized but is important to reveal the detailed mode of action in order to explain the observed synergism. A22 was firstly introduced as an ATP-competitor that binds to the nucleotide binding site of MreB, inhibiting the protein's ATP-dependant polymerization [33]. Later, reports based on structural and dynamic simulations proposed that A22 binds to the A22-binding pocket of MreB, which is located near to the nucleotide binding site, impeding ATP hydrolysis [28]. According to most recent research, A22 disrupts the rod shape of bacterial cells by impeding the ATP-dependent polymerization of MreB in multiple ways [27]. Strahl et al. have reported that MreB protein organizes the bacterial cell membrane and is important for the distribution of membrane proteins [55]. Thus, by targeting MreB, A22 impedes membrane-associated processes including cell membrane organization, cell wall synthesis, and cell wall morphogenesis. The observed synergism between A22 and antibiotics might be due to the damaging of the cell wall by A22, resulting in enhanced membrane permeability, facilitating and increasing the access of antibiotics to their intracellular targets. Thus, further investigation of the molecular interaction between A22 and antibiotics is essential to explain these synergistic effects.

Although MIC and FICI determinations are the standard methods for studying antimicrobial and synergistic activities of single or combined agents, respectively, these techniques provide only evidence about the growth-inhibitory effect of the tested antibiotics [4]. As growth-inhibitory effects are mechanistically distinct from killing effects [56], it was feasible to further investigate if the synergistic interactions exert bacteriostatic or lethal activity by time-kill curve analyses [57]. In monotherapy treatment, sub-MICs of A22 inhibited bacterial growth for a short-time period, while higher concentrations were either bacteriostatic (*E. coli*) or bactericidal (*P. aeruginosa*). The highest tested concentration (2 × MIC) exerted bactericidal activity within 3 h and 24 h against *P. aeruginosa* and *E. coli*, respectively. Similar to our study, A22 has been reported to present a dose-dependent bactericidal activity against the *P. aeruginosa PAO1* standard strain [37]. As it was expected, the results of the combinational time-kill assays showed great synergistic bactericidal interactions with all expected combinations at 24 h of combined treatment.

Biofilm-related infections caused by MDR Gram-negative bacteria have emerged as a major clinical concern. Bacterial cells in biofilm communities are highly resistant to antimicrobials and host immune responses and are therefore difficult to eliminate. Hence, there is an urgent need to develop novel antimicrobials with potent antibiofilm activity, acting in synergy with conventional drugs, targeting both biofilm formation and disruption [9, 11]. The results of the biomass assay in the present study indicated a reduced production of biofilm in both *P. aeruginosa* and *E. coli* isolates when treated with sub-MIC A22, resulting in a lower percentage of viable cells within the matrix compared with the untreated controls. Our findings are in line with a previous study, reporting the inhibitory effect of A22 on *P. aeruginosa* biofilm formation [38]. The ability of A22 to inhibit biofilm formation is probably based on the spherical phenotype cells acquired after the treatment. Cell shape has been reported to play a cruel role in adhesion and biofilm development. Rod-shaped bacteria have enhanced ability to colonize surfaces compared with spherical-shaped cells by maximizing contact area with the substratum [58].

Previous studies that indicated A22 as a potent antibiofilm agent had generally focused on its efficacy to prevent the initial cell adhesion and biofilm formation. However, the removal activity of A22 toward mature preformed biofilms has not been well characterized. Our results for the first time showed the eradication effects of A22 on preformed biofilms of *P. aeruginosa* and *E. coli* clinical isolates. A22 at only the MIC in both strains was able to reduce 80% cell viability of mature biofilms. The majority of the conventional drugs showed a poor biofilm inhibition and eradiation effect at sub-MICs. At combinational studies, CLSM analysis indicated a great biomass reduction of the combination-treated biofilms compared with that of untreated controls. The loosening of architecture and the biomass disruption of preformed biofilms by A22 possible enhanced the entry of conventional drugs within the matrix, resulting in synergistic antibiofilm effects.

The dispersal efficacy of A22 toward preformed biofilms is likely to be controlled by several mechanisms. The formation, severity, and stability of a biofilm depends on many factors, including cell shape, cell death, cell adhesion, and quorum-sensing (QS)-mediated cell motility [14]. Biofilm disruption by A22 might be due to the changes in the morphology of biofilm cells after the treatment. It is well documented that alternations in cell shape caused by antibiotics resulted in major biofilm disruption effects [59]. During the last step of the biofilm growth cycle, many biofilm cells detached from the extracellular matrix to resume a planktonic form of life and colonize new areas [60]. We found that A22 exerted bactericidal activity at 24 h against *P. aeruginosa* and *E. coli* planktonic cells, while the eradiation efficacy of A22 was measured on mature preformed biofilms after 24 h treatment. In that way, A22 may act as a contributor to the native dispersion by killing the cells that dissociate from the biofilm, resulting in overall biofilm loss and preventing further colonization. Another explanation that could be given based on the effect of A22 on the QS-dependant motility complexes. QS-dependant swarming and swimming motility of bacteria plays an important role during cell attachment, biofilm development, and dispersion [14]. It has been reported that the bacterial cytoskeletal proteins, especially MreB, play a fundamental role in cell communication by regulating QS signaling [61]. Notably, previous studies suggested that A22 could inhibit the swimming and swarming motility in *P. aeruginosa PAO1* and *Myxococcus xanthus* [26, 38].

As the aim of this study was to investigate synergistic interaction between A22 and antibiotics, the cytotoxic and hemolytic profile of those combinations had to be tested. Bonez et al. (2016) have reported low cytotoxic and genotoxic activity of A22 monotherapy toward human PBMC cells [37]. However, there were no reports about the hemolytic effects. In our study, we did not observe any hemolytic activity of A22 at the concentrations required to kill or inhibit bacterial cells and biofilms, respectively. A >50% reduction in PMBC and PMN viability was observed only for the concentration of 64 *μ*g/mL (which is the highest MIC against bacteria). We examined the hemolytic and cytotoxic properties of the combinations found to be most synergistic against bacteria. High synergism indicates low FICI values, which means the MICs of the individual compounds are much lower in combination than in monotherapy. At the synergistic combinations, the MIC value of A22 was reduced at least 4-fold to concentrations that are noncytotoxic at all (<32 *μ*g/mL). It is noteworthy that when A22 was combined with antibiotics at concentrations that have been identified as antimicrobial and antibiofilm synergistic, no enhancement of the cytotoxic and hemolytic effect was observed. Overall, the antibacterial, antibiofilm, and cytotoxic combination studies indicated that synergism permits dose reductions of individual drugs and consequently cannot increase the overall cytotoxicity.

The present study concluded that A22 exhibits synergistic antibacterial and antibiofilm properties with low cytotoxic and hemolytic effects. Our data confirm the antimicrobial activity of A22 against clinical isolates of *P. aeruginosa* and expand our knowledge on its activity against clinical *E. coli* strains. The present study also provides new insights into the potential of MreB as a target for novel therapeutics designed to prevent and disrupt biofilm formation. To the best of our knowledge, this is the first study reporting synergistic, noncytotoxic, and nonhemolytic interactions of an MreB inhibitor with various antibiotics, with antibacterial and antibiofilm activities against Gram-negative clinical isolates. These findings provide new evidence that MreB may take a place as a novel antibiotic target not only for single but also for combined antimicrobial therapy and suggest A22 as a compound with desirable properties to study these interactions. Further *in vitro* and *in vivo* studies are required to find out the safety and effectiveness of A22 and other MreB inhibitors in combination with antibiotics in order to introduce them as potential therapeutic options for combined antimicrobial chemotherapy in the future.

## 5. Conclusions

The findings of the present study support previous research regarding the use of MreB as a novel antibiotic target for single or combined antimicrobial chemotherapy in the future. The obtained data expand the existing knowledge about the MreB inhibitor A22 hydrochloride by evaluating the combined antibacterial and antibiofilm activity of the compound with various conventional antibiotics against MDR clinical isolates, as well as the combined cytotoxic and hemolytic activities toward human cells. The findings of the antibacterial assays strongly demonstrate that A22 enhanced the efficacy of the majority of the conventional drugs against planktonic cells, resulting in synergistic effects. Antibiofilm assays revealed great synergism between A22 and CAZ, MERO, CFX, AZM; thus all the tested combinations were able to inhibit biofilm formation at low concentrations and to eradicate mature biofilms formed by clinical pathogens. A22 either as a single agent or in combination showed a minimal cytotoxic activity against human PBMC and PMN cells, and selective hemolytic properties toward human erythrocytes. Overall, these findings strengthen the opinion that MreB protein may be used as a target for the development of novel antimicrobials and indicate A22 as a promising compound for generating new analogs acting in synergy with clinical antibiotics. Further studies are required to investigate the molecular interactions between A22 and conventional drugs and interpret the observed synergism. In addition, further *in vitro* and *in vivo* experiments are necessary to ensure the combined efficacy of A22 and to select specific anti-MreB compounds with desirable properties and minimal cytotoxicity for further development.

## Figures and Tables

**Table 1:** Antimicrobial resistance profile and minimum inhibitory concentration ranges (MIC ranges) of A22 hydrochloride and antibiotics against a total of 68 *P. aeruginosa* and *E. coli* strains.

Strains/antimicrobials	MIC range (*μ*g/mL)	CLSI interpretation^∗^		
		*S* % (*n*)	*I* % (*n*)	*R* % (*n*)
*P. aeruginosa (n = 31)*				
A22	2–64	—	—	—
AMK	2–128	45 (14)	7 (2)	48 (15)
CAZ	4–>256	23 (7)	35 (11)	42 (13)
CIP	0.25–64	32 (10)	3 (1)	65 (20)
CL	0.5–32	74 (23)	—	26 (8)
GEN	1–≥16	58 (18)	10 (3)	32 (10)
MERO	0.5–256	6 (2)	13 (4)	81 (25)

*E. coli (n* *=* *37)*				
A22	4–64	—	—	—
A/S	4/2–128/64	5 (2)	—	95 (35)
AMK	2–128	57 (21)	8 (3)	35 (13)
AZM	4–64	11 (4)	—	89 (33)
CFX	4–≥64	38 (14)	8 (3)	54 (20)
CIP	0.007–>32	16 (6)	5 (2)	79 (29)
MERO	0.06–≥16	60 (22)	5 (2)	35 (13)

^∗^CLSI breakpoints for *P. aeruginosa* susceptible and resistant to amikacin were ≤16 and ≥64, to ceftazidime ≤8 and ≥32, to ciprofloxacin ≤1 and ≥4, to colistin ≤2 and ≥4, to gentamicin ≤4 and ≥16, and to meropenem ≤2 and ≥8 *μ*g/mL, respectively; for *E. coli* susceptible and resistant to ampicillin-sulbactam were ≤8/4 and ≥32/16, to amikacin ≤16 and ≥64, to azithromycin ≤16 and ≥32, to cefoxitin ≤8 and ≥32, to ciprofloxacin ≤1 and ≥4, and to meropenem ≤1 and ≥4 *μ*g/mL, respectively. There is no any susceptibility breakpoint on A22 for any bacteria. *n*: number of strains, *S*: susceptible, *I*: intermediate, *R*: resistant. Antibiotics abbreviations: amikacin (AMK), ampicillin/sulbactam (A/S), azithromycin (AZM), cefoxitin (CFX), ceftazidime (CAZ), colistin (CL), ciprofloxacin (CIP), gentamicin (GEN), meropenem (MERO).

**Table 2:** Interpreted FICI ranges of A22 hydrochloride in combination with antibiotics against 15 clinical strains of *P. aeruginosa* and *E. coli*.

Strains/combinations		FICI range	Synergism % (*n*)	Additive/Indifference % (*n*)	Antagonism % (*n*)
*P. aeruginosa (n* *=* *15)*					
A22+	CAZ	0.5–1	87 (13)	13 (2)	0 (0)
	MERO	0.5–1.5	80 (12)	20 (3)	0 (0)
	GEN	0.5–1.5	73 (11)	27 (4)	0 (0)
	CIP	0.375–1	53 (8)	47 (7)	0 (0)
	AMK	0.5–2	13 (2)	87 (13)	0 (0)
	CL	0.375–2	7 (1)	93 (14)	0 (0)

*E. coli (n* *=* *15)*					
A22+	CFX	0.25–0.5	100 (15)	0 (0)	0 (0)
	AZM	0.18–0.75	93 (14)	7 (1)	0 (0)
	CIP	0.5–1	33 (5)	67 (10)	0 (0)
	AMK	0.5–3	13 (2)	87 (13)	0 (0)
	MERO	0.375–1.5	7 (1)	93 (14)	0 (0)
	A/S	0.5–2	7 (1)	93 (14)	0 (0)

FICI: fractional inhibitory concentration index, *n*: number of strains. Antibiotics abbreviations: amikacin (AMK), ampicillin/sulbactam (A/S), azithromycin (AZM), cefoxitin (CFX), ceftazidime (CAZ), colistin (CL), ciprofloxacin (CIP), gentamicin (GEN), meropenem (MERO).

**Table 3:** Δlog_10_ colony changes of *P. aeruginosa* and *E. coli* strains obtained by the time-kill assay at different time points after treatment with sub-MIC to 2 x MICs of A22, in relation to the initial inoculum.

Strains	Fold MIC	Colony changes (Δlog_10_ cfu/mL) vs. initial inoculum at			
		3 h	6 h	9 h	24 h
*P. aeruginosa*	0.25	+0.96 (⦰)	+0.39 (⦰)	+0.87 (⦰)	+5.41 (⦰)
	0.5	+0.14 (⦰)	−0.54 (Bs)	−0.97 (Bs)	+3.23 (⦰)
	1	−2.36 (Bs)	−3.05 (Bc)	−3.12 (Bc)	−5 (Bc)
	2	−5.04 (Bc)	−5.04 (Bc)	−5.04 (Bc)	−5.04 (Bc)

*E. coli*	0.25	+0.63 (⦰)	+0.7 (⦰)	+0.97 (⦰)	+3.63 (⦰)
	0.5	+0.63 (⦰)	+0.49 (⦰)	+0.15 (⦰)	+2.5 (⦰)
	1	+0.37 (⦰)	+0.35 (⦰)	+0.14 (⦰)	−2.34 (Bs)
	2	−0.78 (Bs)	−1.58 (Bs)	−2.36 (Bs)	−4.85 (Bc)

+: increase growth; −: decrease growth; bacteriostatic (Bs), bactericidal (Bc): <3log_10_ and ≥3log_10_ reduction in cfu/mL, respectively, relative to the initial inoculum; (⦰): no effect.

**Table 4:** Δlog_10_ colony changes of *P. aeruginosa* and *E. coli* strains obtained by the time-kill assay, after 24 h treatment with A22, antibiotics alone in relation to the initial inoculum or their combinations in relation to the initial inoculum and the most active agent.

Strains	Monotherapy, combinations	Fold MIC in monotherapy, combination	Colony changes (Δlog_10_ cfu/mL) at 24 h		Interaction
			vs. initial inoculum	vs. most active agent	
*P. aeruginosa*	A22	0.5	+2.88	—	(⦰)
	CAZ	0.5	+3.92	—	(⦰)
	MERO	0.5	+4.2	—	(⦰)
	GEN	0.5	+3.18	—	(⦰)
	CIP	0.5	+1.64	—	(⦰)
	A22 + CAZ	0.5	−3.59	−6.47	Bactericidal/synergism
	A22 + MERO	0.5	−3.84	−6.72	Bactericidal/synergism
	A22 + GEN	0.5	−4.01	−6.89	Bactericidal/synergism
	A22 + CIP	0.5	−4.29	−6.12	Bactericidal/synergism

*E. coli*	A22	1	−0.98	—	Bacteriostatic
	AZM	1	+3.53	—	(⦰)
	CFX	1	+3.9	—	(⦰)
	A22 + AZM	1	−4.63	−3.68	Bactericidal/synergism
	A22 + CFX	1	−4.66	−3.7	Bactericidal/synergism

Antibiotics abbreviations: azithromycin (AZM), cefoxitin (CFX), ceftazidime (CAZ), ciprofloxacin (CIP), gentamicin (GEN), meropenem (MERO); +: increase growth; −: decrease growth; bacteriostatic, bactericidal: <3log_10_ and ≥3log_10_ reduction in cfu/mL, respectively, relative to the initial inoculum at 24 h; synergism: ≥2log_10_ reduction in cfu/mL relative to the most active agent of the combination at 24 h; (⦰): no effect.

**Table 5:** Hemolytic activity of A22 alone and in combination with conventional antibiotics toward human erythrocytes.

Treatment	Concentration (*μ*g/mL)	Mean hemolytic activity (%) ± SD
A22 hydrochloride	256	0.9 ± 0.4
	2–128	0
Azithromycin	2–256	0
Cefoxitin		
Ceftazidime		
Ciprofloxacin		
Gentamicin		
Meropenem		
A22+ azithromycin	128 + 128	0
A22+ cefoxitin		
A22+ ceftazidime		
A22+ ciprofloxacin		
A22+ gentamicin		
A22+ meropenem		

## Data Availability

The data that support the findings of the current study are available from the corresponding author upon reasonable request.

## Abbreviations

A/S: Ampicillin-sulbactam
A22: A22 hydrochloride
AMK: Amikacin
AZM: Azithromycin
CAMHB: Cation-adjusted Mueller-Hinton broth
CAZ: Ceftazidime
CFU: Colony-forming units
CFX: Cefoxitin
CIP: Ciprofloxacin
CL: Colistin
CLSI: Clinical and Laboratory Standards Institute
CLSM: Confocal laser scanning microscopy
CR: Carbapenem-resistant
CV: Crystal violet
DMSO: Dimethyl sulfoxide
DPBS: Dulbecco's phosphate-buffered saline
*E. coli*: *Escherichia coli*
ESBLs: Extended-spectrum beta-lactamases
FBS: Fetal bovine serum
FICI: Fractional inhibitory concentration index
GEN: Gentamicin
MBLs: Metallo-beta-lactamases
MDR: Multidrug resistant
MERO: Meropenem
MHA: Mueller-Hinton agar
MIC: Minimum inhibitory concentration
MICs: Minimum inhibitory concentrations
MTT: 3-(4,5-dimethylthiazol-2-yl)-2,5-diphenyltetrazolium bromide
NCCLS: National Committee for Clinical Laboratory Standards
OD: Optical density
*P. aeruginosa*: *Pseudomonas aeruginosa*
PBMCs: Peripheral blood mononuclear cells
PBS: Phosphate-buffered saline
PMNs: Polymorphonuclear neutrophils
QS: Quorum sensing
RBCs: Red blood cells
SD: Standard deviation
WHO: World Health Organization.

## Supplementary Materials

Supplementary Materials Supplementary Table S1. MIC values of A22 and antibiotic resistance patterns of *P. aeruginosa* isolates. Supplementary Table S2. MIC values of A22 and antibiotic resistance patterns of *E. coli* isolates.
