# Supplementary material for: Synergistic Antibacterial and Antibiofilm Activity of the MreB Inhibitor A22 Hydrochloride in Combination with Conventional Antibiotics against Pseudomonas aeruginosa and Escherichia coli Clinical Isolates
Source: Int J Microbiol. 2021 Aug 25;2021:3057754. doi: 10.1155/2021/3057754 (PMC8413048; doi:10.1155/2021/3057754)
Supplement: Supplementary Materials — Supplementary Table S1. MIC values of A22 and antibiotic resistance patterns of P. aeruginosa isolates. Supplementary Table S2. MIC values of A22 and antibiotic resistance patterns of E. coli isolates. [file 3057754.f1.zip › 3057754.f1/Supplementary Table S1.docx]

| Isolates | Antimicrobial Agents | | | | | | | Number of antibiotics in which resistance was observed |
| --- | --- | --- | --- | --- | --- | --- | --- | --- |
|  | A22 | AMK | CAZ | CIP | CL | GEN | MERO |  |
|  | MIC (μg/mL) | Resistance patterns | | | | | |  |
| NCIMB 12469 | 16 | S | S | S | S | S | S | - |
| Clinical: #1 | 2 | S | S | S | S | S | I | - |
| #2 | 2 | S | I | S | S | S | R | 1 |
| **#3** | 4 | R | I | R | R | R | R | 5 |
| #4 | 4 | S | S | S | S | S | I | - |
| #5 | 4 | S | R | S | S | S | R | 2 |
| #6 | 4 | S | R | S | S | S | R | 2 |
| **#7** | 4 | R | R | R | S | R | R | 5 |
| **#8** | 8 | R | I | R | R | R | R | 5 |
| #9 | 8 | R | I | R | S | S | R | 3 |
| **#10** | 8 | R | I | R | S | I | R | 3 |
| **#11** | 8 | R | I | R | S | I | R | 3 |
| #12 | 8 | S | S | S | S | S | I | - |
| #13 | 8 | S | R | R | S | S | R | 3 |
| #14 | 8 | S | S | S | S | S | R | 1 |
| **#15** | 8 | R | R | R | R | R | R | 6 |
| **#16** | 16 | I | R | R | R | R | R | 5 |
| **#17** | 16 | R | I | R | R | R | R | 5 |
| #18 | 16 | S | I | R | S | S | R | 2 |
| **#19** | 16 | R | R | R | S | R | R | 5 |
| #20 | 16 | R | S | R | S | S | R | 3 |
| **#21** | 16 | R | I | R | S | R | R | 4 |
| **#22** | 16 | R | R | R | S | R | I | 4 |
| #23 | 16 | S | R | S | S | S | R | 2 |
| #24 | 32 | S | S | I | S | S | S | - |
| **#25** | 32 | R | R | R | S | R | R | 5 |
| **#26** | 64 | R | R | R | R | S | R | 5 |
| **#27** | 64 | I | R | R | R | I | R | 4 |
| **#28** | 64 | R | R | R | R | S | R | 5 |
| #29 | 64 | S | I | S | S | S | R | 1 |
| #30 | 64 | S | I | R | S | S | R | 2 |
| A22 MIC range  -  Resistant isolates  % Resistance | 2-64 | 15/31  48 | 13/31  42 | 20/31  65 | 8/31  26 | 10/31  32 | 25/31  81 |  |

**Table S1.** MIC values of A22 and antibiotic resistance patterns of *P. aeruginosa* isolates.

S: susceptible, I: intermediate, R: resistant.

Antibiotics abbreviations: Amikacin (AMK), Ceftazidime (CAZ), Colistin (CL), Ciprofloxacin (CIP), Gentamicin (GEN), Meropenem (MERO).

*Isolates used in checkerboard assays are indicated in bold.
