# Supplementary material for: Synergistic Antibacterial and Antibiofilm Activity of the MreB Inhibitor A22 Hydrochloride in Combination with Conventional Antibiotics against Pseudomonas aeruginosa and Escherichia coli Clinical Isolates
Source: Int J Microbiol. 2021 Aug 25;2021:3057754. doi: 10.1155/2021/3057754 (PMC8413048; doi:10.1155/2021/3057754)
Supplement: Supplementary Materials — Supplementary Table S1. MIC values of A22 and antibiotic resistance patterns of P. aeruginosa isolates. Supplementary Table S2. MIC values of A22 and antibiotic resistance patterns of E. coli isolates. [file 3057754.f1.zip › 3057754.f1/Supplementary Table S2.docx]

**Table S2.** MIC values of A22 and antibiotic resistance patterns of *E. coli* isolates.

| Isolates | Antimicrobial Agents | | | | | | | Number of antibiotics in which resistance was observed |
| --- | --- | --- | --- | --- | --- | --- | --- | --- |
|  | A22 | A/S | AMK | AZM | CFX | CIP | MERO |  |
|  | MIC (μg/mL) | Resistance patterns | | | | | |  |
| NCIMB 8879 | 32 | S | S | S | S | S | S | - |
| Clinical: #1 | 4 | R | R | S | I | R | S | 3 |
| #2 | 4 | R | S | S | R | R | S | 3 |
| #3 | 4 | R | S | R | R | R | S | 4 |
| **#4** | 4 | R | R | R | R | R | S | 5 |
| #5 | 4 | S | S | S | S | S | S | - |
| #6 | 4 | R | S | R | S | I | R | 3 |
| **#7** | 4 | R | R | R | R | R | R | 6 |
| #8 | 4 | R | I | R | S | R | S | 3 |
| #9 | 4 | R | I | R | S | R | S | 3 |
| #10 | 4 | R | S | R | S | R | S | 3 |
| #11 | 4 | R | R | R | S | R | S | 4 |
| **#12** | 4 | R | R | R | R | R | S | 5 |
| #13 | 8 | R | R | R | S | R | I | 4 |
| #14 | 8 | R | S | R | S | S | I | 2 |
| **#15** | 8 | R | S | R | R | R | R | 5 |
| #16 | 8 | R | S | R | S | S | R | 3 |
| #17 | 8 | R | R | R | I | R | S | 4 |
| **#18** | 8 | R | R | R | R | I | R | 5 |
| #19 | 8 | R | S | R | R | S | R | 4 |
| **#20** | 16 | R | S | R | I | R | R | 4 |
| **#21** | 16 | R | S | R | R | R | R | 5 |
| **#22** | 16 | R | S | R | R | R | R | 5 |
| #23 | 16 | R | S | R | R | S | R | 4 |
| **#24** | 16 | R | R | R | R | R | R | 6 |
| **#25** | 16 | R | R | R | R | R | S | 5 |
| #26 | 32 | R | S | R | R | R | S | 4 |
| **#27** | 32 | R | R | R | R | R | S | 5 |
| **#28** | 32 | R | R | R | R | R | S | 5 |
| #29 | 32 | R | R | R | S | R | S | 4 |
| #30 | 32 | R | S | R | R | R | S | 4 |
| **#31** | 64 | R | S | R | R | R | S | 4 |
| #32 | 64 | R | S | R | S | R | S | 3 |
| **#33** | 64 | R | I | R | R | R | R | 5 |
| #34 | 64 | R | S | R | R | R | S | 4 |
| #35 | 64 | R | S | R | S | R | S | 3 |
| **#36** | 64 | R | S | R | S | R | R | 4 |
| A22 MIC range  -  Resistant isolates  % Resistance | 4-64 | 35/37  95 | 13/37  35 | 33/37  89 | 20/37  54 | 29/37  79 | 13/37  35 |  |

S: susceptible, I: intermediate, R: resistant.

Antibiotics abbreviations: Amikacin (AMK), Ampicillin/Sulbactam (A/S), Azithromycin (AZM), Cefoxitin (CFX), Ciprofloxacin (CIP), Meropenem (MERO).

*Isolates used in checkerboard assays are indicated in bold.
